# The pro-inflammatory marker soluble suppression of tumorigenicity-2 (ST2) is reduced especially in diabetic morbidly obese patients undergoing bariatric surgery

**DOI:** 10.1186/s12933-020-01001-y

**Published:** 2020-02-26

**Authors:** Svitlana Demyanets, Christoph Kaun, Alexandra Kaider, Walter Speidl, Manfred Prager, Stanislav Oravec, Philipp Hohensinner, Johann Wojta, Gersina Rega-Kaun

**Affiliations:** 1 Department of Laboratory Medicine, Medical University of Vienna, Waehringer Guertel 18-20, 1090 Vienna, Austria; 2 Department of Internal Medicine II, Division of Cardiology, Medical University of Vienna, Waehringer Guertel 18-20, 1090 Vienna, Austria; 3 Center for Medical Statistics, Informatics, and Intelligent Systems, Medical University of Vienna, Waehringer Guertel 18-20, 1090 Vienna, Austria; 4 Department of Surgery, Hospital Hietzing, Wolkersbergenstraße 1, 1130 Vienna, Austria; 5 Krankenanstalten Dr. Dostal, Saarplatz 9, 1190 Vienna, Austria; 6 2nd Department of Internal Medicine, Faculty of Medicine, Comenius University, Šafárikovo námestie 6, 814 99 Bratislava 1, Slovakia; 7 Core Facilities, Medical University of Vienna, Waehringer Guertel 18-20, 1090 Vienna, Austria; 8 Ludwig Boltzmann Institute for Cardiovascular Research, Waehringer Guertel 18-20, 1090 Vienna, Austria; 9 5th Medical Department, Wilhelminenhospital, Montleartstraße 37, 1160 Vienna, Austria

**Keywords:** sST2, Obesity, Diabetes, Bariatric surgery

## Abstract

**Background:**

High soluble suppression of tumorigenicity-2 (sST2) is a marker of poor prognosis in chronic inflammatory conditions. ST2 and its ligand interleukin (IL)-33 are elevated in adipose tissue of obese individuals. We aimed to evaluate circulating sST2 and IL-33 as possible markers of metabolic benefit in morbidly overweight patients after Roux-en-Y gastric bypass (RYGB) bariatric surgery.

**Methods:**

sST2, IL-33, high sensitive IL-6, high sensitive C-reactive protein (hsCRP), leptin, cholesterol metabolism and liver parameters were measured in 80 morbidly obese individuals before and 1 year after bariatric surgery.

**Results:**

sST2 was higher (P = 0.03) in diabetics as compared to individuals without diabetes. Baseline sST2 was also higher in males than in females (P= 0.0002). One year after bariatric surgery, sST2 levels were decreased (median 120, IQR 59–176 pg/mL) as compared to sST2 before surgery (median 141, IQR 111–181, P = 0.0024), and the diabetic group showed most pronounced reduction in sST2 (P = 0.0016). An association was found between sST2 and liver function parameters before and after bariatric surgery, and between baseline sST2 and total cholesterol, triglyceride, total low density lipoprotein (LDL), small dense LDL, Apolipoprotein B as well as with small dense high density lipoproteins (HDL). In the subgroup of diabetic patients positive correlation between IL-33 and sST2 (r = 0.44, P = 0.05) was noticed.

**Conclusions:**

Circulating sST2 is associated with markers of liver functions and lipid metabolism in severely obese patients and a reduction of sST2 was shown after successful bariatric surgery, most prominently in diabetic patients.

## Background

Obesity became a global health-threatening problem, leading to the development of associated complications such as insulin resistance, type 2 diabetes, cardiovascular disease, and liver disease. Obesity is accompanied by chronic low-grade inflammation that plays an etiological role in the development of metabolic dysregulation [1–3].

Transmembrane suppression of tumorigenicity-2 (ST2L) is the receptor for interleukin (IL)-33, a cytokine that belongs to the IL-1 family [4–6]. Both ST2L and soluble ST2 (sST2) as well as IL-33 are expressed in many tissues including adipose tissue and are increased in obesity [7–9]. In murine models in the setting of obesity, IL-33 exerts protective effects by reducing adiposity and improving glucose and insulin tolerance [8, 10, 11]. sST2 is a decoy receptor for free IL-33, which prevents action of IL-33 by its binding and neutralization [12]. Circulating levels of sST2 are an established marker for prognosis in patients with coronary artery disease, heart failure, and even in critically ill patients [13–17].

Data on circulating sST2 in the setting of human obesity and diabetes are rare. Levels of sST2 were increased in severely obese individuals [9] and higher sST2 levels were also associated with the presence of diabetes mellitus in different study cohorts [18–21].

Bariatric surgery is an optional treatment for severe obesity [22], which was shown to be associated with reduced long term mortality especially in diabetic patients [23]. Weight loss due to bariatric surgery is associated with increased insulin sensitivity and reduced markers of inflammation such as high-sensitive C-reactive protein (hsCRP) and IL-6 [24, 25], reduced markers of vascular dysfunction such as thrombomodulin and E-selectin [26] as well as reduced pro-inflammatory senescence-associated secretory proteins and extracellular vesicles from the liver as was shown by us recently [27, 28].

However, data on the dynamic of sST2 levels before and after bariatric surgery are not available. The IL-33/sST2 axis seems to play a role in metabolic disorders, but this is mostly based on in vitro studies and on in vivo studies performed in animals [8, 10, 11]. Therefore, we aimed to investigate the effect of bariatric surgery on sST2 serum levels in morbidly obese patients. In addition, we compared serum levels of sST2 in individuals with normal glucose tolerance (NGT), prediabetes and diabetes and its relations to other metabolic parameters.

## Methods

### Design, sampling and ethical approval

80 study participants were consecutively enrolled after they were selected to undergo gastric bypass surgery fulfilling suggested criteria [29]. This included a body mass index (BMI) > 40 kg/m^2^ or a BMI > 35 kg/m^2^ with secondary disease, a minimum age of 18 years and the failure or futility of a structured conservative program. Exclusion criteria were other bariatric surgery procedures than Roux-en-Y gastric bypass (RYGB) surgery, acute infection, cancer, or any other consuming disease.

The study protocol was approved by the ethics committee of Burgenland and by the ethics committee of the Medical University of Vienna. We hereby confirm that all methods were performed according to the guidelines and regulations approved by the local ethics committee and to the guidelines of the Declaration of Helsinki. Written informed consent was obtained from each participant in the study. Participation in the study was voluntary and patients could withdraw their consent at any time.

Subjects were divided as having NGT, prediabetes or diabetes according to the guidelines of the American Diabetes Association [30]. Baseline characteristics of the cohort are presented in Table 1. Follow up visits for physical examination and blood collection were arranged 12 months after surgery in 62 individuals. Before surgery and at follow up visit a venous blood drawing was performed. After centrifugation (2800 r.p.m., 20 min), plasma and serum samples were aliquoted and stored at − 80 ℃.

**Table 1:** Characteristics of the subjects at baseline examination

Variable	All subjects N = 80
Age, years, mean (SD)	40.8 (12.7)
Sex, male, n (%)	24 (30.0%)
Weight, kg, mean (SD)	128.7 (18.3)
Body-mass index, kg/m^2^, mean (SD)	44.2 (3.9)
Waist circumference, cm, mean (SD)	134.1 (10.1)
Cardiovascular risk factors, n (%)	
Preexisting cardiovascular disease	1 (1.3%)
Hypertension	37 (46.3%)
Type 2 diabetes mellitus	25 (31.3%)
Prediabetes (IGT or IFG)	14 (17.5%)
Current cigarette use	33 (41.3%)
Hyperlipidemia	20 (25.0%)
Current therapy, n (%)	
Statins	18 (22.5%)
Oral glucose lowering agents	18 (22.5%)
Insulin	5 (6.3%)
Blood pressure lowering agents	37 (46.3%)
Angiotensin converting enzyme-inhibitors	32 (40.0%)
Beta-blockers	19 (23.8%)

*IGT* impaired glucose tolerance, *IFG* impaired fasting glucose

### Measurements of sST2, IL-33, hsIL-6 and leptin

Serum concentration of sST2 and IL-33 was assessed by specific commercially available enzyme-linked immunosorbent assays (ELISA) as described by us previously [14, 15, 31]. sST2 was quantified using human ST2/IL-1 R4 DuoSet^®^ ELISA Kit and IL-33 was measured using human IL-33 DuoSet ELISA (both R&D Systems, Minneapolis, MN, USA). Serum leptin and hsIL-6 concentrations were measured by specific ELISAs (Mercodia Leptin ELISA (Mercodia Inc., Uppsala, Sweden) and hsIL-6 ELISA (R&D Systems), respectively).

### Laboratory parameters

Glucose, hsCRP, aspartat-aminotransferase (ASAT, GOT), alanin-aminotransferase (ALAT, GPT), and gamma-glutamyl-transferase (GGT) were measured under standardized conditions in an ISO 15189 accredited medical laboratory on Cobas 8000 analyzer (Roche Diagnostics, Mannheim, Germany). N-terminal pro-brain natriuretic peptide (NT-proBNP) was analyzed in the same laboratory on Cobas e 411 analyzer (Roche Diagnostics). Glycated haemoglobin A1(HbA1c) was determined by high-performance liquid chromatography (HPLC) separation of hemoglobin fractions.

Lipoprotein spectrum with lipoprotein subpopulations were analyzed and quantitatively evaluated by Quantimetrix Lipoprint LDL system (Quantimetrix Corporation, Redondo Beach, CA, USA), an electrophoresis method on polyacrylamide gel (PAG) [32, 33]. Total cholesterol and triglycerides in serum were analyzed by an enzymatic method CHOD PAP (Roche Diagnostics).

### Statistical analysis

Median values (and interquartile ranges (IQR)) are given to describe the continuous variables. Variables with right-skewed distributions were log-transformed prior to statistical analyses. The Pearson correlation coefficient was calculated to describe the association between laboratory parameters and sST2 levels, and the Spearman correlation coefficient was used to evaluate the associations with serum IL-33 concentrations. Correlation coefficients (r) lower than − 0.3 or higher than 0.3 were considered as clinically relevant. The paired t-test was calculated to analyze the change in sST2 in the first year after bariatric surgery within the individual patients. Since log-transformed sST2 values were considered for statistical analyses, the size of the changes is depicted by the geometric mean ratio (GMR), resulting from retransforming the mean difference of the logarithmic scale. Analysis of variance (ANOVA) models were performed to test for statistically significant differences between groups of patients with respect to sST2 levels at two time points (baseline and 1 year after surgery). An interaction term was included in the ANOVA models to evaluate whether changes in the sST2 levels due to bariatric surgery differ between the patient groups. An analysis of covariance (ANCOVA) model was performed to assess the influence of age and sex on the sST2 values at the two different time points, and an interaction term was included to test for an age-dependent gender difference. Two-sided P-values less than 0.05 were considered statistically significant. The software SAS (version 9.4, SAS Institute Inc. (2016); Cary, NC, USA) was used for all statistical analyses.

## Results

### sST2 is decreased after bariatric surgery

**Fig. 1:**
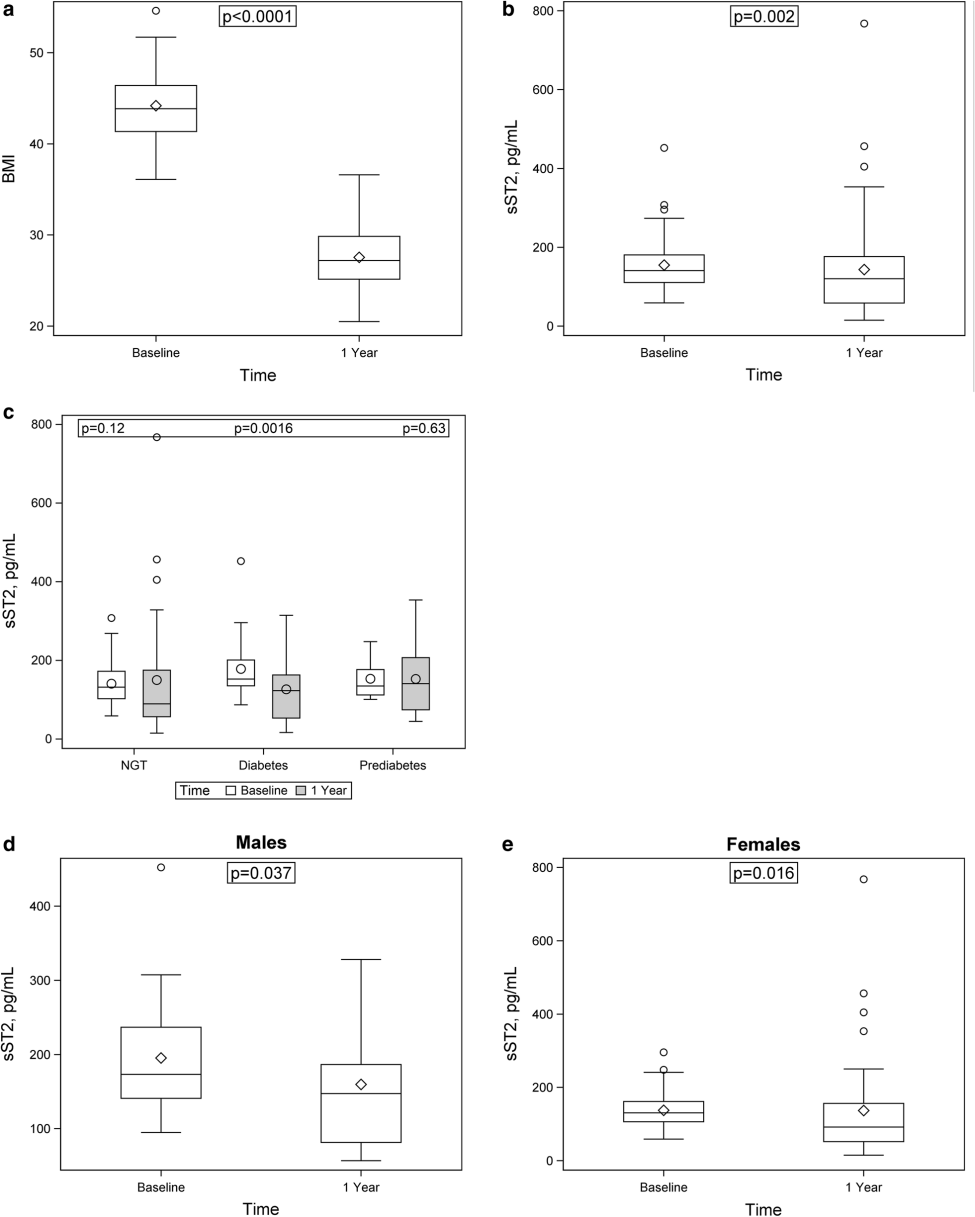
Weight and sST2 concentrations are decreased after bariatric surgery. Box-whisker plot showing BMI (**a**) and serum sST2 concentrations (pg/mL) before and 1 year after bariatric surgery (**b**–**e**) and stratified according to the presence of diabetes (**c**) and gender (**d**, males and **e**, females). sST2 was determined in samples obtained before and 1 year after bariatric surgery as indicated in Methods. NGT: normal glucose tolerance

One year after bariatric surgery obese individuals drastically lost weight (P < 0.0001, Fig. 1a). sST2 levels also significantly decreased one year after surgery (median 120, IQR 59–176 pg/mL, n = 62) as compared to sST2 levels before bariatric surgery (median 141, IQR 111–181 pg/mL, P = 0.0024, n = 80, Fig. 1b). Overall, the serum level of sST2 was reduced by 27% in the entire cohort (GMR 0.73; 95% confidence interval (CI) 0.60–0.89). When sST2 levels were determined before and after bariatric surgery in diabetics, in prediabetic individuals and in individuals with NGT, only in the group of diabetics the decrease of sST2 by 43% reached significance (baseline: median 153, IQR 136–201 pg/mL; 1 year after surgery: median 123, IQR 53–163 pg/mL, P = 0.0016) with a GMR 0.57 (95%CI 0.42–0.78). In individuals with prediabetes (baseline: median 135, IQR 112–177 pg/mL; 1 year after surgery: median 141, IQR 74–207 pg/mL, P = 0.63) or with NGT (baseline: median 132, IQR 103–172 pg/mL; 1 year after surgery: median 90, IQR 57–175 pg/mL, P = 0.12, Fig. 1c) no significant changes in sST2 levels could be observed. The individual longitudinal changes in sST2 in the entire cohort of obese individuals and in the subgroup of diabetic patients are shown in Additional file 1: Fig. S1A and B, respectively. Serum levels of IL-33 1 year after surgery (median 335, IQR 8–1574 pg/mL) tended to be higher compared with baseline levels (median 226, IQR 10–1369 pg/mL) without reaching significance (P ≥ 0.05, Additional file 1: Fig. S2). In both, males (baseline: median 173, IQR 141–237 pg/mL; 1 year after surgery: median 147, IQR 81–187 pg/mL, P = 0.037, Fig. 1d) and females (baseline: median 131, IQR 106–162 pg/mL; 1 year after surgery: median 92, IQR 52–156 pg/mL, P = 0.016, Fig. 1e), sST2 levels significantly decreased 1 year after bariatric surgery with no evidence that one sex profits more than the other from the surgery (P = 0.78).

### Relation of baseline sST2 to the metabolic profile

**Fig. 2:**
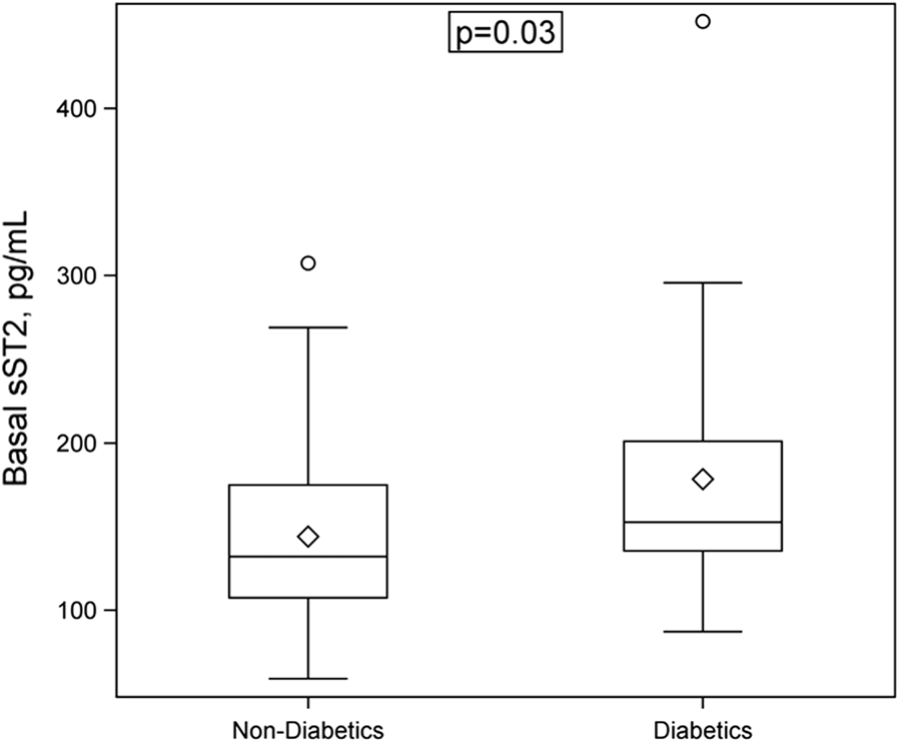
Levels of sST2 are higher in obese individuals with diabetes. Box-whisker plot showing basal serum sST2 concentrations (pg/mL) stratified according to the presence of diabetes. sST2 was determined in samples obtained before bariatric surgery as indicated in Methods

When patients were divided in two groups with diabetes (median 153, IQR 136–201 pg/mL, N = 25) and without diabetes (median 132, IQR 107–175 pg/mL, N = 55), latter included subjects with NGT and prediabetes, sST2 levels were significant higher in diabetics (P = 0.03, Fig. 2).

Basal sST2 showed moderate positive associations with liver function parameters ALAT (r = 0.41, P = 0.0002), GOT (r = 0.33, P = 0.003), and GGT (r = 0.38, P = 0.0006) (Table 2, Additional file 1: Figs. 3A–C). Diabetic patients showed strong positive correlation between basal sST2 and GPT (r = 0.60, P = 0.002), ASAT (r = 0.52, P = 0.008), and GGT (r = 0.63, P = 0.0008), respectively. In subjects with NGT only GPT remained significantly associated with sST2 (r = 0.36, P = 0.02), and liver parameters showed no relevant correlation with sST2 in prediabetes individuals. Therefore, correlations between sST2 and liver function parameters seen in the entire cohort at baseline seem to be mainly attributed to the diabetic patients.

**Table 2:** Correlation of sST2 with metabolic parameters before and after surgery in the entire cohort

	sST2 before surgery		sST2 after surgery	
	r value	P value	r value	P value
Total cholesterol	0.44	< 0.0001	< 0.3	n.s.
Triglyceride	0.42	< 0.0001	< 0.3	n.s.
Total LDL	0.36	0.001	< 0.3	n.s.
Small dense LDL	0.38	0.0006	< 0.3	n.s.
Apolipoprotein B	0.44	< 0.0001	< 0.3	n.s.
Small dense HDL	0.35	0.002	< 0.3	n.s.
Total HDL	< 0.3	n.s.	< 0.3	n.s.
Apolipoprotein A1	< 0.3	n.s.	< 0.3	n.s.
GPT	0.41	0.0002	0.42	0.0008
GOT	0.33	0.003	0.44	0.0005
GGT	0.38	0.0006	0.47	0.0001
Leptin	− 0.31	0.006	− 0.34	0.006
Glucose	< 0.3	n.s.	< 0.3	n.s.
HbA1c	< 0.3	n.s.		
BMI	< 0.3	n.s.	< 0.3	n.s.
Weight	< 0.3	n.s.	< 0.3	n.s.
Waist circumference	< 0.3	n.s.	< 0.3	n.s.
hsCRP	< 0.3	n.s.	< 0.3	n.s.
hsIL-6	< 0.3	n.s.	0.32	0.01

*LDL* low density lipoproteins, *HDL* high density lipoproteins, *GPT* alanin-aminotransferase, *GOT* aspartat-aminotransferase, *GGT* gamma-glutamyl-transferase, *HbA1c* glycated hemoglobin, *BMI* body mass index, *hsCRP* high-sensitive C-reactive protein, *hsIL-6* high-sensitive interleukin-6

Basal sST2 was associated with lipid parameters such as total cholesterol (r = 0.44, P < 0.0001), triglyceride (r = 0.42, P < 0.0001), total low density lipoprotein (LDL) (r = 0.36, P = 0.001), small dense LDL (r = 0.38, P = 0.0006), Apolipoprotein B (r = 0.44, P < 0.0001) as well as with small dense high density lipoproteins (HDL) (r = 0.35, P = 0.002, Additional file 1: Figs. 3D–I) but not with total HDL or Apolipoprotein A1 (both |r| < 0.3, P > 0.05) (Table 2).

No correlations were noticed between basal sST2 and glucose, HbA1c, BMI, weight, waist circumference, NT-proBNP, hsCRP or hsIL-6 (all |r| < 0.3, P > 0.05). However, sST2 showed a weak negative correlation with leptin levels (r = − 0.31, P = 0.006, Table 2).

Only diabetic patients demonstrated positive correlation between sST2 and IL-33 (r = 0.44, P = 0.05), although such correlation was not evident in the entire cohort or in individuals with NGT or prediabetes. Serum IL-33 showed no correlation with liver function parameters (all |r| < 0.3, P > 0.05).

### Relation of sST2 to the metabolic profile 1 year after bariatric surgery

1 year after surgery associations between sST2 and liver function parameters GPT (r = 0.42, P = 0.0008), GOT (r = 0.44, P = 0.0005), and GGT (r = 0.47, P = 0.0001) remained relevant in the entire cohort (Table 2). These associations were strongest in the NGT group (sST2 and GPT: r = 0.53, P = 0.002; sST2 and GOT: r = 0.54, P = 0.002; sST2 and GGT: r = 0.59, P = 0.0007), weaker in the diabetes group (sST2 and GPT: r = 0.39, P > 0.05; sST2 and GOT: r = 0.39, P > 0.05; sST2 and GGT: r = 0.36, P > 0.05), and almost not evident in the prediabetes group (sST2 and GPT: |r| < 0.3, P > 0.05; sST2 and GOT: |r| < 0.3, P > 0.05; sST2 and GGT: r = 0.39, P > 0.05).

Concerning the associations of sST2 with different lipid parameters, 1 year after surgery, no associations of sST2 with total cholesterol, triglyceride, total LDL, small LDL or Apolipoprotein B were observed (all |r| < 0.3, P > 0.05, Table 2).

Similarly to baseline, one year after surgery sST2 was not associated with BMI, weight, waist circumference or serum hsCRP (all |r| < 0.3, P > 0.05) but showed similar weak negative correlation with leptin levels (r = − 0.34, P = 0.006, Table 2). Moreover, sST2 did not correlate with glucose levels in the entire cohort nor in the study subgroups (all |r| < 0.3, P > 0.05). Only 12 months sST2 values correlated weakly positive with hsIL-6 (r = 0.32, P = 0.01, Table 2), which was not the case in the basal blood samples, as described above. The positive association between hsIL-6 and sST2 was seen only in NGT group (r = 0.49, P = 0.006), but not in the diabetes group (|r| < 0.3, P > 0.05) and in contrast to a weak negative correlation in the prediabetes group (r = − 0.33, P > 0.05).

No correlation was noticed between sST2 and IL-33 in the entire cohort nor in the study subgroups 1 year after bariatric surgery.

### Relation of sST2 to gender and age

**Fig. 3:**
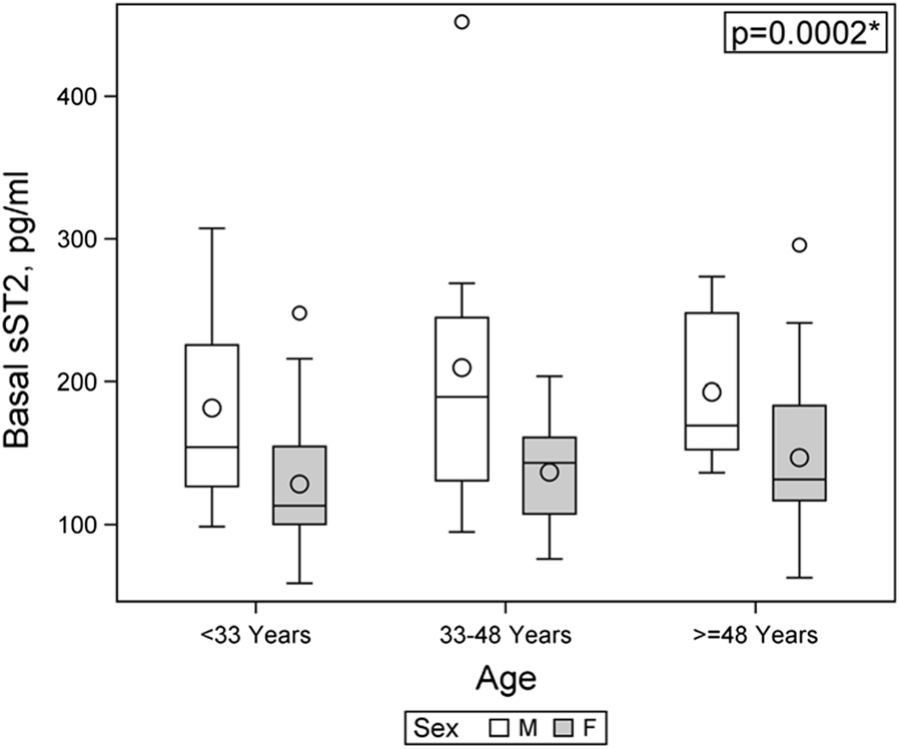
Baseline sST2 concentrations are higher in male obese individuals. Box-whisker plot showing basal serum sST2 concentrations (pg/mL) stratified according to age and gender. sST2 was determined in samples obtained before bariatric surgery as indicated in Methods

In our cohort, baseline sST2 concentrations were higher in males (N = 24, median 173, IQR 141–237 pg/mL) than in females (N = 56; median 131, IQR 106–162 pg/mL, P = 0.0002, Fig. 3). This difference remained significant after adjusting for age (P = 0.0002). The trend in gender sST2 difference was observed also 1 year after surgery (males: median 147, IQR 81–187 pg/mL vs females: median 92, IQR 52–156 pg/mL, P = 0.076). The trend in gender difference remained after age-adjustment (P = 0.076).

## Discussion

We provide direct comparison of circulating levels of sST2, which is a decoy receptor for IL-33, in obese patients with different metabolic states before and 1 year after bariatric surgery. Overall, sST2 levels are decreased in morbidly obese patients after bariatric surgery. Interestingly, sST2 decrease was more pronounced in the group of diabetic patients. Furthermore, basal sST2 levels were higher in diabetics compared to obese individuals without diabetes. In line with previous results [20], male obese individuals demonstrated higher sST2 levels as female obese individuals. Moreover, we showed that before surgery sST2 is associated with liver enzymes and lipid profile mostly in diabetic obese patients. In contrast, 1 year after bariatric surgery the association between sST2 and liver function parameters was seen only in NGT group and the association between sST2 and lipid parameters was not evident anymore.

Bariatric surgery, recently also referred to as metabolic surgery [34], is in general associated not only with weight loss but also with improved metabolic health and down-regulation of pro-inflammatory mediators [22, 27, 35]. Interestingly, absence of diabetes led to a greater decrease in total cholesterol, LDL-C, and non-HDL-C [36]. On the other hand, however, weight gain after diabetes diagnosis was associated with higher mortality [37].

Zeyda et al. found elevated ST2 and IL-33 expression in omental and subcutaneous adipose tissue as well as increased plasma sST2 in severely obese subjects as compared to lean controls [9]. Gleimer et al. also revealed higher sST2 in obese patients than in normal weight patients among children and adults undergoing hematopoietic cell transplantation [38]. However, the dynamic of circulating sST2 as well as its correlation with clinical and laboratory parameters before and after bariatric surgery was not studied before.

Our results showing that obese individuals with diabetes exhibit higher sST2 levels than obese persons without diabetes correspond to the previous reports in other cohorts, which, however, included mostly non-obese individuals. Association of sST2 with diabetes was demonstrated in individuals largely without vascular disease, including Framingham Heart Study [19] and pSoBid cohort [20], patients with left ventricular diastolic dysfunction [18], and heart failure [39]. Circulating levels of sST2 were associated with diabetic markers such as hepatic function, triglycerides, and plasma glucose [18, 20]. Moreover, higher levels of sST2 were associated with complications of diabetes such as development of diabetic nephropathy [40] or critical limb ischemia, where its levels predict mortality [41].

It could be speculated that different organs including heart and adipose tissue are responsible for sST2 levels, measured in the circulation in humans. The proportion of sST2 produced by each single organ or tissue would most probably depend on the presence or absence of a particular pathological condition. E.g. hypertrophic stimuli were shown previously to stimulate sST2 secretion [42].

Obesity and type 2 diabetes are the leading risk factors for the development of nonalcoholic steatohepatitis (NASH) [43]. The IL-33/ST2 pathway was shown to have a profibrotic role in an experimental model of diet-induced NASH [44]. Similar to Miller et al. [20], we found a clear association between sST2 and liver enzymes GPT, GOT, and GGT in our cohort. Strictly, although before surgery this association was driven by the correlation in the subgroup of diabetics, 12 months after surgery such associations were seen only in the individuals with normal glucose tolerance.

Dyslipidemia is a hallmark of obesity and type 2 diabetes [45]. Previous reports showed an association between sST2 and total cholesterol and HDL-cholesterol [21] as well as triglycerides [20] in non-obese patients even if the role of IL-33/ST2 system in the regulation of lipid homeostasis is still not well determined [46]. Our study here is the first to investigate the relationship between sST2 and different lipid subtypes before and after bariatric surgery. We found a significant association between basal sST2 and total cholesterol, triglyceride, total LDL, small dense LDL, Apolipoprotein B and small dense HDL.

Previous publications consistently showed higher sST2 levels in male than in female participants [19, 20, 39, 47]. We observed that also in morbidly obese individuals, sST2 levels were higher in males than in females. Although, the underlying mechanisms for gender-specific differences in sST2 levels are still unknown [48], our results support the importance to adjust sST2 levels for sex and probably to apply different reference values if studying this biomarker.

The following limitations of our study have to be considered. Even if our study population included a well-characterized population of morbidly obese patients, deviation in the number of individuals in the subgroups before and after bariatric surgery due to loss of follow-up could influence our results. A thorough validation of the sST2 dynamics at different time pointes, also at longer follow up, after bariatric surgery in individuals with healthy and unhealthy obesity and different co-morbidities has to be performed to confirm and complement our results. Moreover, the correlations of sST2 levels with established metabolic-related blood parameters are weak to moderate in our study cohort. Furthermore, an analysis of IL-33/ST2 expression in different fat depots would be desirable but could not be performed in our study. Therefore, it is too early to propose sST2 as a complimentary routine blood marker in morbidly obese individuals. However, despite these limitations, our finding strengthens the potential for the use of the pro-inflammatory marker sST2 in morbidly obese especially diabetic patients to determine the metabolic state as well as to monitor the success of bariatric surgery.

## Conclusion

We demonstrated tight relationships of sST2 with diabetes, liver function parameters and lipid profile in obese individuals and showed that bariatric surgery may have an impact on these associations.

## Supplementary information

**Additional file 1: Fig. S1.** Individual longitudinal changes in sST2 levels. Individual sST2 levels (log transformed) before and one year after bariatric surgery in the entire cohort (A) and in diabetic patients (B). Red lines show mean changes in sST2. **Fig. S2.** Serum IL-33 concentrations before and after bariatric surgery. Box-whisker plot showing serum IL-33 concentration (pg/mL) before and one year after bariatric surgery in morbidly obese individuals. **Fig. S3.** Correlation of baseline sST2 with liver enzymes and lipid parameters. Correlations of sST2 with GPT (alanin-aminotransferase, A), GOT (aspartate-aminotransferase, B), GGT (gamma-glutamyl-transferase, C), total cholesterol (D), triglyceride (E), total low density lipoprotein (LDL, F), small dense LDL (G), apoliprotein B (H), and small dense high density lipoprotein (HDL, I) are shown.

## Data Availability

The datasets used and/or analyzed during the current study are available from the corresponding author on reasonable request.

## Abbreviations

ALAT: Alanin-aminotransferase
ANCOVA: Analysis of covariance
ANOVA: Analysis of variance
BMI: Body mass index
CRP: C-reactive protein
ELISA: Enzyme-linked immunosorbent assay
GGT: Gamma-glutamyl-transferase
GMR: Geometric mean ratio
HbA1c: Glycated haemoglobin A1
HDL: High density lipoproteins
HPLC: High-performance liquid chromatography
hs: High sensitive
IL: Interleukin
IQR: Interquartile range
LDL: Low density lipoprotein
NASH: Nonalcoholic steatohepatitis
NGT: Normal glucose tolerance
PAG: Polyacrylamide gel
sST2: Soluble suppression of tumorigenicity-2
ST2L: Transmembrane suppression of tumorigenicity-2
95%CI: 95% confidence interval
