# Supplementary material for: The pro-inflammatory marker soluble suppression of tumorigenicity-2 (ST2) is reduced especially in diabetic morbidly obese patients undergoing bariatric surgery
Source: Cardiovasc Diabetol. 2020 Feb 26;19:26. doi: 10.1186/s12933-020-01001-y (PMC7045735; doi:10.1186/s12933-020-01001-y)
Supplement: Supplementary file 1 — Additional file 1: Fig. S1. Individual longitudinal changes in sST2 levels. Individual sST2 levels (log transformed) before and one year after bariatric surgery in the entire cohort (A) and in diabetic patients (B). Red lines show mean changes in sST2. Fig. S2. Serum IL-33 concentrations before and after bariatric surgery. Box-whisker plot showing serum IL-33 concentration (pg/mL) before and one year after bariatric surgery in morbidly obese individuals. Fig. S3. Correlation of baseline sST2 with liver enzymes and lipid parameters. Correlations of sST2 with GPT (alanin-aminotransferase, A), GOT (aspartate-aminotransferase, B), GGT (gamma-glutamyl-transferase, C), total cholesterol (D), triglyceride (E), total low density lipoprotein (LDL, F), small dense LDL (G), apoliprotein B (H), and small dense high density lipoprotein (HDL, I) are shown. [file 12933_2020_1001_MOESM1_ESM.docx]

**The pro-inflammatory marker soluble suppression of tumorigenicity-2 (ST2) is reduced especially in diabetic morbidly obese patients undergoing bariatric surgery**

Svitlana Demyanets, Christoph Kaun, Alexandra Kaider, Walter Speidl, Manfred Prager, Stanislav Oravec, Philipp Hohensinner, Johann Wojta, Gersina Rega-Kaun

| A | 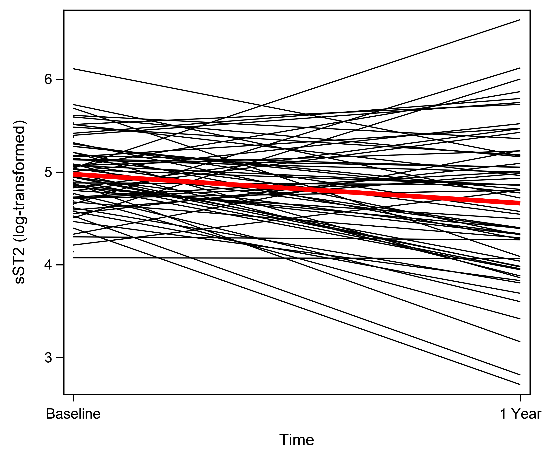 | B | 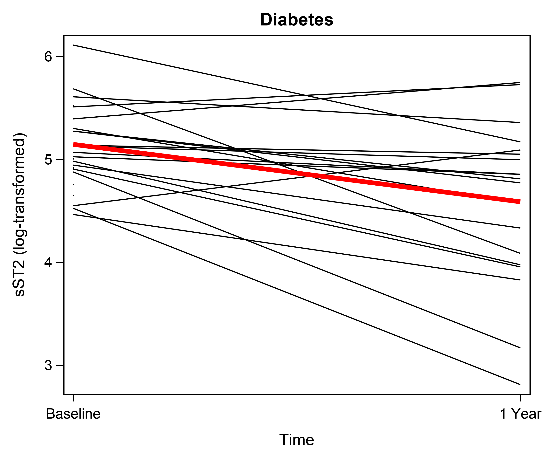 |
| --- | --- | --- | --- |

**Additional file 1 Fig. S1. Individual longitudinal changes in sST2 levels.** Individual sST2 levels (log transformed) before and one year after bariatric surgery in the entire cohort (A) and in diabetic patients (B). Red lines show mean changes in sST2.


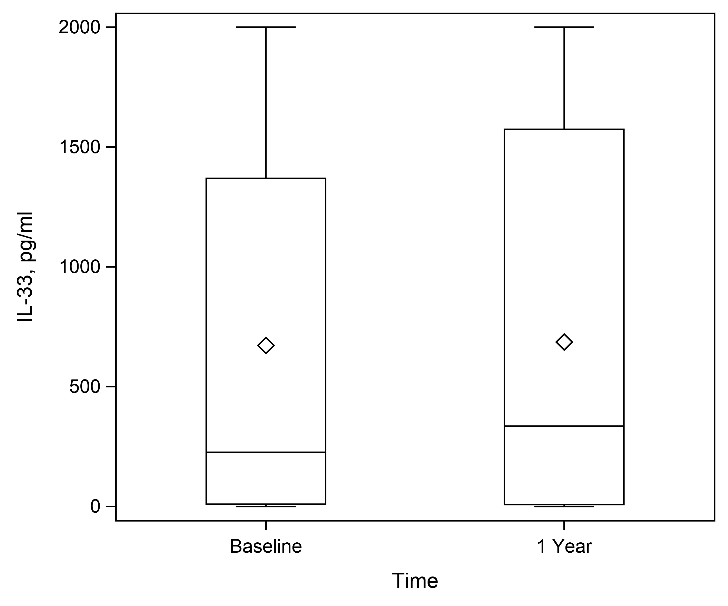


**Additional file 1 Fig. S2. Serum IL-33 concentrations before and after bariatric surgery.** Box-whisker plot showing serum IL-33 concentration (pg/mL) before and one year after bariatric surgery in morbidly obese individuals.

| A | **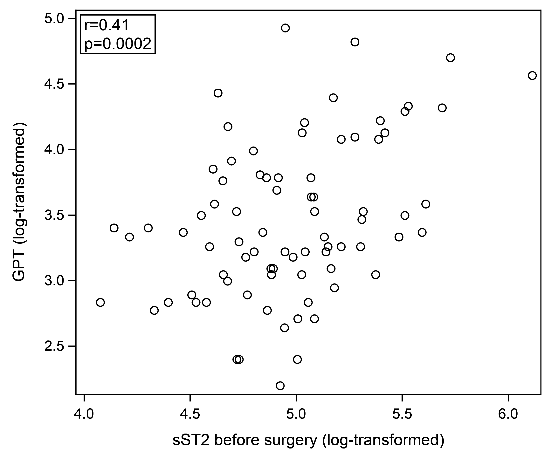** | B | **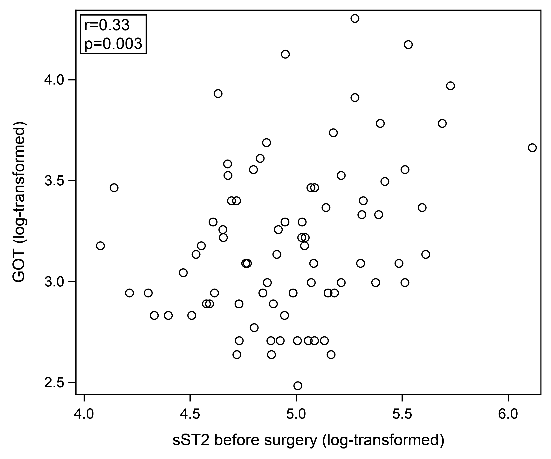** |
| --- | --- | --- | --- |
|  |  |  |  |
| C | **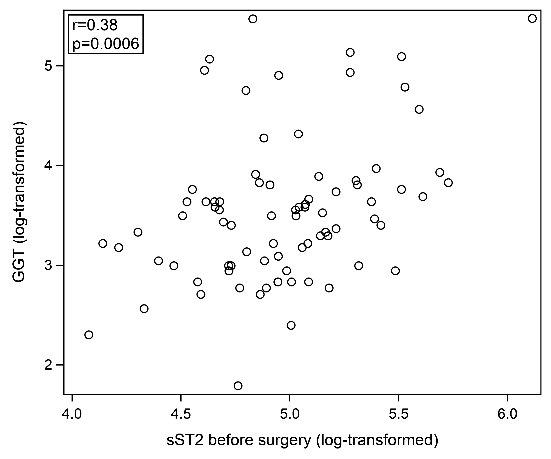** | D | **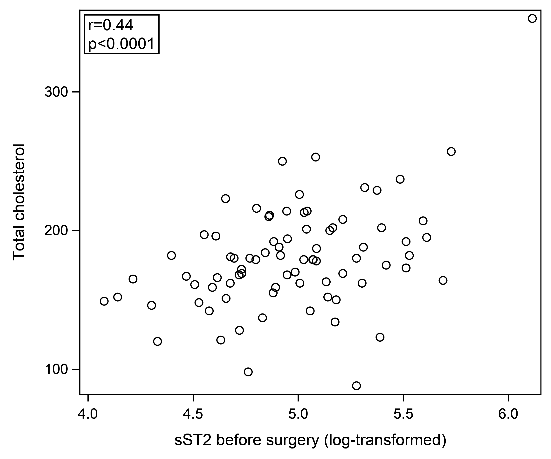** |
|  |  |  |  |
| E | **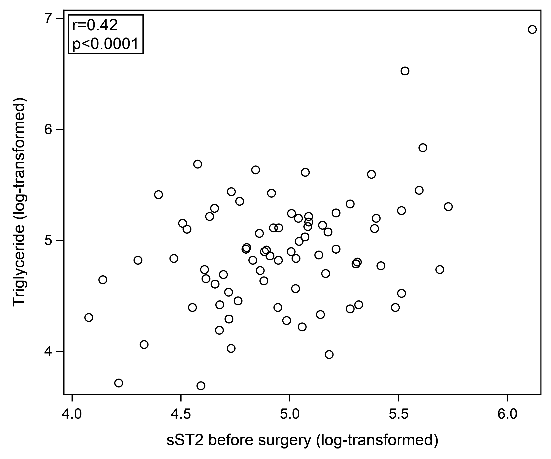** | F | **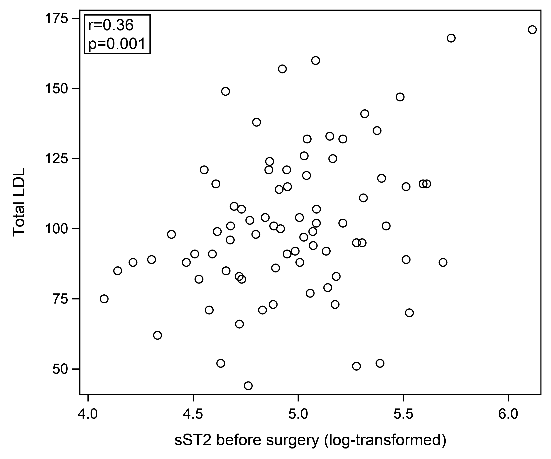** |
|  |  |  |  |
| G | **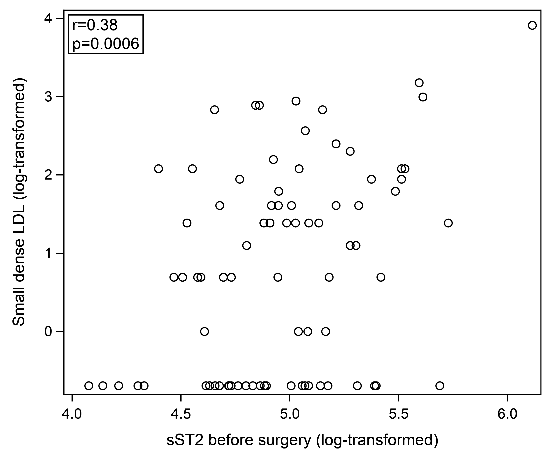** | H | **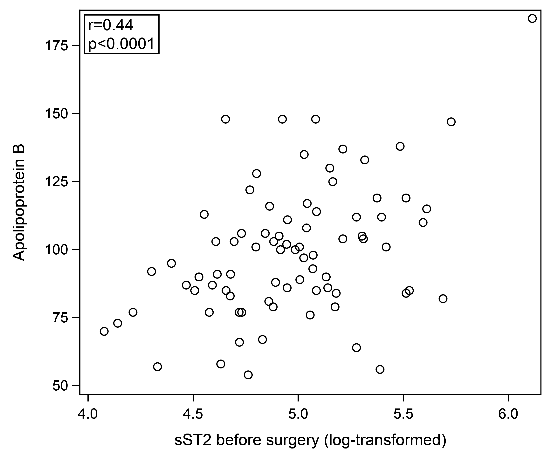** |
|  |  |  |  |
| I | **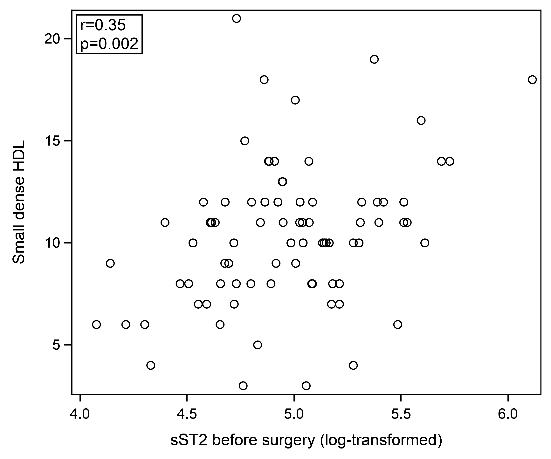** |  |  |

**Additional file 1 Fig. S3. Correlation of baseline sST2 with liver enzymes and lipid parameters.** Correlations of sST2 with GPT (alanin-aminotransferase, A), GOT (aspartate-aminotransferase, B), GGT (gamma-glutamyl-transferase, C), total cholesterol (D), triglyceride (E), total low density lipoprotein (LDL, F), small dense LDL (G), apoliprotein B (H), and small dense high density lipoprotein (HDL, I) are shown.
